# A Novel Bifunctional Wax Ester Synthase Involved in Early Triacylglycerol Accumulation in Unicellular Green Microalga *Haematococcus pluvialis* Under High Light Stress

**DOI:** 10.3389/fbioe.2021.794714

**Published:** 2022-01-17

**Authors:** Haiyan Ma, Jie Zheng, Yanhua Li, Liang Zhao, Song Zou, Qiang Hu, Danxiang Han

**Affiliations:** ^1^ Center for Microalgal Biotechnology and Biofuels, Institute of Hydrobiology, Chinese Academy of Sciences, Wuhan, China; ^2^ University of Chinese Academy of Sciences, Beijing, China; ^3^ Institute for Advanced Study, Shenzhen University, Shenzhen, China; ^4^ Laboratory for Marine Biology and Biotechnology, Qingdao National Laboratory for Marine Science and Technology, Qingdao, China; ^5^ State Key Laboratory of Freshwater Ecology and Biotechnology, Institute of Hydrobiology, Chinese Academy of Sciences, Wuhan, China; ^6^ Key Laboratory for Algal Biology, Institute of Hydrobiology, Chinese Academy of Sciences, Wuhan, China

**Keywords:** triacylglycerol, wax synthase, microalgae, bifunctional, *Haematococcus pluvialis*, biotechnological application

## Abstract

The bulk of neutral lipids, including astaxanthin esters and triacylglycerols (TAGs), are accumulated in the green microalga *Haematococcus pluvialis* under high light (HL) stress. In this study, a novel bifunctional wax ester synthase (WS) gene was cloned from *H. pluvialis* upon HL stress. The overexpression of HpWS restored the biosynthesis of wax esters and TAGs in neutral lipid-deficient yeast mutant *Saccharomyces cerevisiae* H1246 fed with C18 alcohol and C18:1/C18:3 fatty acids, respectively. Under HL stress, *HpWS* was substantially upregulated at the transcript level, prior to that of the type I diacylglycerol:acyl-CoA acyltransferase encoding gene (*HpDGAT1*). HpDGAT1 is the major TAG synthase in *H. pluvialis*. In addition, the application of xanthohumol (a DGAT1/2 inhibitor) in the *H. pluvialis* cells did not completely eliminate the TAG biosynthesis under HL stress at 24 h. These results indicated that HpWS may contribute to the accumulation of TAGs in *H. pluvialis* at the early stage under HL stress. In addition, the overexpression of HpWS in *Chlamydomonas reinhardtii bkt5*, which is engineered to produce free astaxanthin, enhanced the production of TAGs and astaxanthin. Our findings broaden the understanding of TAG biosynthesis in microalgae and provide a new molecular target for genetic manipulation in biotechnological applications.

## Introduction

Photosynthetic microalgae accumulate neutral lipids mainly in the form of triacylglycerols (TAGs) under unfavorable conditions, such as nutrient limitation, high temperature, and high light (HL) intensities (Chisti, 2007; Hu et al., 2008; Georgianna and Mayfield, 2012). In addition to acting as an energy storage pool, TAG molecules play the role of signaling molecules and are involved in many physiological processes of the photosynthetic organisms (Yang and Benning, 2018). For oleaginous microalgae, TAGs stored under nitrogen deprivation can reach 20%–50% of dry weight (Hu et al., 2008). Fast-growing oleaginous microalgal species have been considered as promising cell factories to produce lipids and other high-value products (Radakovits et al., 2010).

Scientists have dissected the biosynthesis of TAGs in some microalgae (Li-Beisson et al., 2015; Zienkiewicz et al., 2016). There are two major TAG biosynthesis pathways: Kennedy and monoacylglycerol pathways. In either pathway, diacylglycerol (DAG) is converted to TAGs by an acyl-CoA:DAG acyltransferase (DGAT), which is the committed step of TAG biosynthesis (Yen et al., 2008). In addition, TAGs can be synthesized by phospholipid:DAG acyltransferase (PDAT) in the acyl-CoA independent pathway. DAG, phospholipid, and galactolipid act as acyl donors, and DAG acts as acyl acceptor (Yoon et al., 2012). It has been reported that multifunctional acyltransferases can synthesize TAGs in some plant and algae species. In *Arabidopsis thaliana*, two phytyl ester synthases (PESs), PES1 and PES2, can employ acyl-CoAs and galactolipids as acyl donors to synthesize phytyl ester and TAGs (Lippold et al., 2012). In diatom *Phaeodactylum tricornutum*, a dual-function PtWS/DGAT was identified to synthesize wax ester (WEs) and TAGs. The dual-function PtWS/DGAT was considered to be a promising target for genetic engineering in the microalgal-based lipid industry (Cui et al., 2018). MOBAT with wax synthase (WS) and DGAT activity was characterized in *Chromochloris zofingiensis* (Xu et al., 2021).

Notably, in some plant and microalgal species, such as *Simmondsia chinensis* and *Euglena gracilis*, WEs can be accumulated as the major energy and storage carbon (Lardizabal et al., 2000; Teerawanichpan and Qiu, 2010). Similar to TAGs, WEs also perform essential physiological functions such as stress response in plants and microalgae (Lewandowska and Keyl, 2020). WSs perform the acylation of fatty alcohol (Lardizabal et al., 2000; Teerawanichpan and Qiu, 2010). There are three WS families—plant-like WS, bifunctional WS/DGAT (WSD), and mammalian WS—which are closely related to or overlap with the DGATs (Röttig and Steinbüchel, 2013). Plant-like WS and mammalian WS are phylogenetically grouped with the MBOAT family (DGAT1) and the DGAT2/acyl-CoA:monoacylglycerol acyltransferase family, respectively. WSD is also considered as one type of DGAT (Liu et al., 2012).

TAG is a valuable product from microalgae in the biofuel industry. Thus, many studies have been focused on improving and re-designing the TAG biosynthesis pathway in the potential oleaginous species through genetic engineering approaches (Radakovits et al., 2010). The primary and popular strategy for increasing the TAG content and designing the desired TAG profile is to directly overexpress the key enzymes from the fatty acid (FA) biosynthesis or TAG assembly pathway (Dehesh et al., 2001; Yamaoka et al., 2016; Xin et al., 2017; Mao et al., 2019; Xin et al., 2019; Haslam et al., 2020).

The green microalga *Haematococcus pluvialis* can produce a broad spectrum of lipids, with potential as a biodiesel feedstock (Damiani et al., 2010; Han et al., 2013). Under the stress condition, especially in HL, *H. pluvialis* cells synthesized bulk of neutral lipids, including astaxanthin ester, TAGs, and sterol esters (SEs) stored in lipid bodies (LBs) (Lee and Zhang, 1999; Boussiba, 2000). Coordination of astaxanthin and FA biosynthesis was proposed in *H. pluvialis*, and DGAT was suspected as an acyltransferase for the synthesis of astaxanthin esters (Chen et al., 2015). Interestingly, under HL exposure, a similar molecular-level coordination was observed between sterol and FA in *H. pluvialis* (Scodelaro Bilbao et al., 2020). The DGATs in *H. pluvialis* have been characterized and functionally interpreted, and HpDGAT1 was proposed to be the major TAG synthase (Ma et al., 2020). However, the expression of *HpDGAT1* was upregulated late under HL stress. This indicates that there may be another TAG synthase besides HpDGAT1.

In this study, a novel bifunctional plant/algae WS enzyme was cloned from *H. pluvialis*. The overexpression of HpWS restored the TAG and WE biosyntheses in neutral lipid-deficient yeast mutant *Saccharomyces cerevisiae* H1246 by extra feeding with appropriate fatty alcohol and FA species. The expression of *HpWS* that responded to HL exposure was earlier and faster than that of *HpDGAT1*. This indicates that *HpWS* was involved in TAG accumulation in *H. pluvialis* at the early stage under stress. Overexpression of WS in *Chlamydomonas reinhardtii bkt5*, an engineering strain capable of producing free astaxanthin, enhanced the production of oils and astaxanthin. Our findings broaden the understanding of TAG biosynthesis in microalgae in response to stresses and provide a new target for genetic manipulation in biotechnological applications.

## Materials and Methods

### Strains and Culturing Conditions

The algal species, *H. pluvialis* NIES144, was obtained from the National Institute for Environmental Studies (Tsukuba, Japan). Algal cells were maintained at 20°C with a light intensity of 20 μmol m^−2^ s^−1^ in 100 ml of basal medium (Kobayashi et al., 1991) under a 12/12-h light/dark cycle. Upon HL stress, the cells were cultured and sampled as in our previous study (Ma et al., 2020). The algal species *C. reinhardtii bkt5* was generously donated by Prof. Matteo Ballottari from the University of Verona (Perozeni et al., 2020). The strain was maintained on the Tris-Acetate-Phosphate (TAP) agar plate with paromomycin (2 μg ml^−1^) at 25°C with a light intensity of 30–50 μmol m^−2^ s^−1^.

### Cloning of HpWS

With the RNA-seq analysis, a transcript of the putative membrane-bound *O*-acyltransferase (MBOAT) family gene was continuously upregulated during HL stress (Ma et al., 2020). The full-length cDNA sequence of *HpWS* gene was obtained by Rapid Amplification of cDNA Ends (RACE) (Ma et al., 2020). The primers used for RACE are shown in Supplementary Table S1. The orthologs of WS, DGAT, PES, and WSD families from various organisms were applied for phylogenetic analysis. The alignment of the amino acid (AA) was performed by ClustalW, and the phylogenetic tree was constructed using the maximum likelihood method (1,000 bootstrap test) based on JTT matrix-based model (MEGA 7.0 software). The transmembrane domains of interested proteins were predicted using TMHMM (http://www.cbs.dtu.dk/services/TMHMM/).

### Functional Analysis of HpWS in *Saccharomyces cerevisiae* H1246

The primers used to construct pYES2-HpWS vectors are listed in Supplementary Table S1. The expression of recombinant HpWS and lipid extraction were performed according to previous studies (Chen et al., 2015; Ma et al., 2020) with slight modifications. The induction temperature was at 25°C instead of 30°C. To check protein expression, the membrane pellet was re-suspended in a mixture of 60 μl of buffer A (0.1 M of DTT and 0.1 M Na_2_CO_3_) and 40 μl of buffer B (30% sucrose and 5% sodium dodecyl sulfate (SDS)) and was vortexed for 30 min at room temperature. Insoluble protein was removed at 12,000 *g* for 10 min at 4°C. The protein concentration in the supernatant was measured with a CB-X™ protein assay kit (G Biosciences, St. Louis, MO, USA). The proteins (10 μg in each sample) were separated by SDS–polyacrylamide gel electrophoresis (SDS-PAGE) on a 10% precast polyacrylamide gel (Bio-Rad, Hercules, CA, USA) and transferred to a polyvinylidene difluoride (PVDF) membrane (Bio-Rad). The antibody against His-tag (HRP-66005, ProteinTech, Chicago, IL, USA) was introduced to check the expression of HpWS. The lipid residue was re-established in chloroform, and the volume was normalized to OD600. Long-chain fatty alcohols and FAs were separately dissolved in hot ethanol as stocks and applied to the yeast during induction with the final concentration of 0.1% (*w*/*v*). The WEs and TAGs were separated by thin-layer chromatography (TLC), and *n*-hexane/diethyl ester/acetate acid (90:7.5:1) and petroleum ether/diethyl ether/acetic acid (80:20:1) elution were used as developing solvent, respectively. Trioleate, arachidyl dodecanoate, dioleate glycerol, and oleic acid were introduced as standards for TAG, WE, DAG, and free FA (FFA), respectively. The produced neutral lipids spots were visualized by spraying with CuSO_4_/H_3_PO_4_ followed by charring. The relative content of produced lipid species was quantified *via* ImageJ software.

### Gene Expression Patterns of HpWS and HpDGATs and Neutral Lipids Accumulation in *Haematococcus pluvialis* Under High-Light Stress

*H. pluvialis* NIES144 was cultured in a 250-ml Erlenmeyer flask containing 100 ml of basal medium with the light intensity of 30 μmol m^−2^ s^−1^ at 20°C. When the cell density reached 3 × 10^5^ cell ml^−1^, the cultures were exposed to HL stress (195 μmol m^−2^ s^−1^). The cultures grown under the light intensity of 30 μmol m^−2^ s^−1^ were set as control groups. For RNA extraction, 1.5 ml of cell culture was taken at 0, 3, 6, 12, 24, and 48 h after HL induction. In addition, 10 ml of cell culture was taken for pigment and TAG quantification at 0, 24, and 48 h after HL induction. Algal cells were harvested by centrifuging at 1,500 *g* for 10 min at room temperature. The pellets were frozen in liquid nitrogen and stored at −80°C before use. The experiment was performed in three biological replicates.

Gene expression was quantified by reverse transcription–quantitative PCR (RT-qPCR) techniques. RNA was extracted, and the first-strand cDNA was synthesized (Ma et al., 2020). The internal reference gene used was 18S rRNA. The primers that were used to amplify genes and internal references are listed in Supplementary Table S1. The relative fold change in expression was calculated as 2^−ΔΔCt^ (Livak and Schmittgen, 2001).

For pigment extraction, the frozen pellet was transferred to a 2-ml screw-top vial containing 200 μl of glass beads (acid-washed, Sigma-Aldrich, St. Louis, MO, USA). The cells were resuspended in 500 μl of dichloromethane/methanol (v:v = 1: 3) and disrupted using the Mini-beadbeater (2.5 × 10^3^ oscillations per minute for 1 min). The disrupted cells were immediately cooled on ice for 1 min. After being centrifuged at 12,000 *g* for 10 min at 4°C, the supernatant was carefully transferred to a new 2-ml centrifuge tube. The remaining pellet was extracted two more times until the color of the cell pellet turned pale. The supernatant was combined and was dried under N_2_. The residue was re-established in dichloromethane/methanol (v:v = 1: 3), and the volume was normalized to cell number. The free astaxanthin and astaxanthin esters in the extracts were analyzed and quantified by high-performance liquid chromatography (HPLC) (Li et al., 2010).

Lipid was extracted as described above. The lipid residue was re-established in chloroform, and the volume was normalized to cell number. An equal volume of each sample was loaded on TLC for separation. The SEs and TAGs were separated by TLC. Cholesteryl palmitate was introduced as a standard for SEs. The produced TAG and SE spots visualized by iodine vapor staining were scraped from the plate and transferred to a 2-ml brown glass vial. The methyl esterification of TAGs was performed (Wu et al., 2019).

### Identification of Wax Ester and Sterol Ester

For the identification of WEs, the scrapped samples from TLC were extracted twice with 500 μl of *n*-hexane. The organic solvent was transferred to a new glass vial and dried under N_2_. The residue was re-dissolved in 200 μl of *n*-hexane and analyzed directly by gas chromatography (GC). The samples were analyzed on an Agilent 7890B GC unit (Agilent Technologies, Santa Clara, CA, USA) with a flame ionization detector (FID). The separation was carried out on an HP-5MS column (30 m × 0.25 mm × 0.25 μm). The carrier gas was nitrogen with a constant flow rate of 1 ml min^−1^. One microliter of the sample was injected in a splitless mode in an injector maintained at 250°C. The chromatographic separation was initially set at 80°C (held for 1 min), raised by 10°C min^−1^ to 230°C (held for 2 min), and then raised by 4°C min^−1^ to 300°C (held for 8 min). The FID temperature was set to 300°C. Arachidyl dodecanoate was introduced as the standard.

For the identification of SE, the scrapped sample from TLC was heated in 300 μl of methanol (including 7.5% KOH) at 80°C for 2 h. After being cooled to room temperature, the released free sterol was extracted by 1 ml of *n*-hexane. Sterols were derivatized by heating at 80°C for 2 h with the presence of BSTFA (1% TMCS, Sigma-Aldrich). The free sterols in native and derivatized form were analyzed by both GC and GC–mass spectrometry (GC-MS). The samples were analyzed on an Agilent 7890B GC coupled with 5977A mass spectrometer. The separation was carried out on an HP-5MS column (30 m × 0.25 mm × 0.25 μm). The carrier gas was helium with a constant flow rate of 1 ml min^−1^. One μl of the sample was injected in a splitless mode in an injector maintained at 250°C. The chromatographic separation was initially set at 150°C (held for 1 min) and raised by 10°C min^−1^ to 300°C (held for 11 min). In the GC-MS detection, the spectrometry was set to scan in the range of *m*/*z* 50–600 at 70 eV with electron impact (EI) ionization mode.

### Effect of DGAT Inhibitors on Triacylglycerol Production in *Haematococcus pluvialis* Under High-Light Stress

*H. pluvialis* NIES144 was cultured in a 250-ml Erlenmeyer flask containing 100 ml of basal medium with the light intensity of 30 μmol m^−2^ s^−1^ at 20°C. When the cell density reached 3 × 10^5^ cell ml^−1^, the cultures were exposed to HL stress (195 μmol m^−2^ s^−1^). The DAGT1-specific inhibitor A922500 was added to cultures at the final concentration of 7.5, 15, and 30 μM (HA groups). Xanthohumol, an inhibitor of both DGAT1 and DGAT2, was added to cultures at the final concentration of 5, 10, and 40 μM (HX groups). A922500 and xanthohumol have been found to inhibit the activity of DGATs in the *in vitro* assay (Zhao et al., 2008; Inokoshi et al., 2009). The HL group without inhibitors was introduced as the control group. After 24 h of exposure to HL, 5 ml of cell culture was sampled for TAG quantification. The algal cells were harvested by centrifugation at 1,500 *g* for 10 min at room temperature, and TAGs were extracted and analyzed. The experiment was performed in three biological replicates. The expression of five copies of *HpDGAT* along with *HpWS* was determined in the samples with 30 μM of A922500 or 40 μM of xanthohumol. The relative gene expression was calculated as 2^−ΔΔCt^. The primers that were used to amplify HpDGATs and internal references are listed in Supplementary Table S1.

### Overexpression of HpWS in *Chlamydomonas reinhardtii bkt5*

Transformation of *C. reinhardtii bkt*5 with HpWS was performed with slight modifications, according to Ma et al. (2020)and Wu et al. (2019). The cDNA encoding *HpWS* was constructed into the vector pClamy_4 (Invitrogen, Carlsbad, CA, USA) using the primers listed in Supplementary Table S1. The TAP agar plate containing zeocin (2 μg ml^−1^) and paromomycin (5 μg ml^−1^) was used for screening. The primers for determining the expression of *HpWS* and six copies of *DGATs* from *C. reinhardtii* in positive transformants are listed in Supplementary Table S1. The relative gene expression was calculated as 2^−ΔCt^. The internal reference gene used was α-tubulin.

The overexpressing transformant colonies of selected *HpWS* were inoculated in a 50-ml Erlenmeyer flask containing 10 ml of TAP medium at 25°C with the light intensity of 50 μmol m^−2^ s^−1^ for 3 days as seeds. The seeds were then inoculated into a column photobioreactor containing 700 ml of TAP medium, with an initial concentration of 2.5 × 10^5^ cell ml^−1^, and cultivated at 25°C with a light intensity of 200 μmol m^−2^ s^−1^. Each column was supplied with filtered air containing 3% CO_2_. After 3 days, the culture medium was transferred to a nitrogen-deprived TAP medium. The culture was cultured for another 3 days. During the cultivation process, a fixed volume of algae culture was sampled daily to determine the dry weight and cell density. For the pigment and oil quantification, 100 ml of cell culture was sampled daily after switching to the nitrogen-depleted medium. The algal cells were harvested by centrifuging at 1,500 *g* for 10 min at room temperature, and the pellet was lyophilized for 2 days. The algal powder was weighted and extracted for pigment and oil analysis, according to Wu et al. (2019)and Yoon et al. (2012), respectively.

### Data Analysis

All the data were processed with Excel 2010 (Microsoft^®^, Albuquerque, NM, USA) and expressed as mean ± SD. The statistical analysis was performed with Student’s *t*-test, and *p* < 0.05 was considered as statistically significant.

## Results

### Identification and Cloning of HpWS Encoding Gene

**FIGURE 1:**
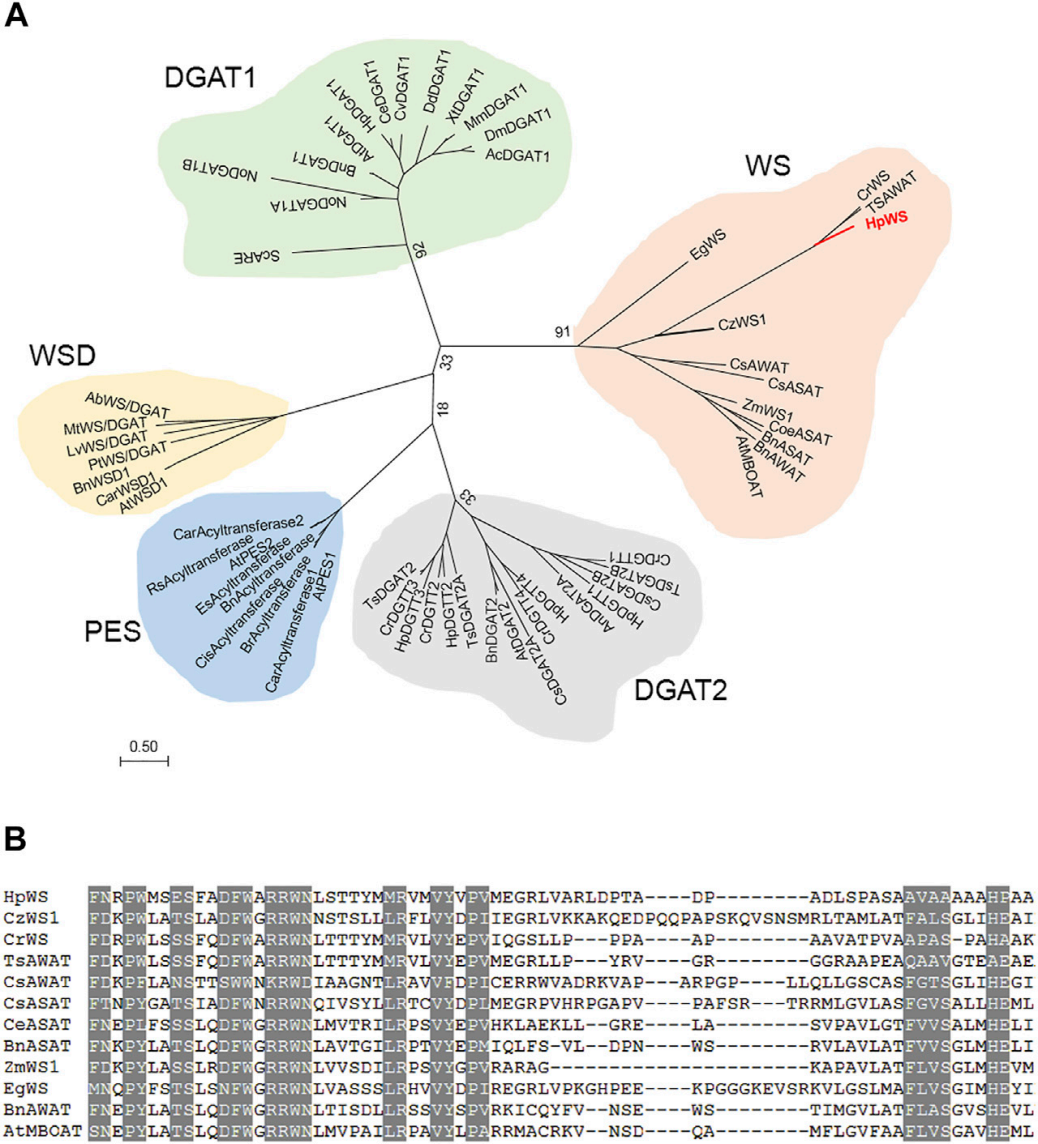
Phylogenetic and alignment analysis of HpWS. **(A)** Phylogenetic tree of wax synthase (WS), diacylglycerol acyltransferase (DGAT), phytyl ester synthase (PES), and bifunctional WS/DGAT (WSD) from various organisms. **(B)** Conserved motifs in WS clade. Ab, *Acinetobacter baylyi*; Ac, *Apis cerana*; At, *Arabidopsis thaliana*; An, *Aspergillus niger*; Bn, *Brassica napus*; Car, *Capsella rubella*; Ce, *Chlamydomonas eustigma*; Cis, *Citrus sinensis*; Coe, *Coffea eugenioides*; Cr, *Chlamydomonas reinhardtii*; Cs, *Chlorella sorokiniana*; Cv, *Chlorella vulgaris*; Cz, *Chromochloris zofingiensis*; Dd, *Dictyostelium discoideum*; Dm, *Drosophila melanogaster*; Eg, *Euglena gracilis*; Es, *Eutrema salsugineum*; Hp, *Haematococcus pluvialis*; Lv, *Leptolyngbya valderiana*; Mm, *Mus musculus*; Mt, *Mycobacterium tuberculosis*; No, *Nannochloropsis oceanica*; Pt, *Phaeodactylum tricornutum*; *Rs*, *Raphanus sativus*; Sc, *Saccharomyces cerevisiae*; Ts, *Tetrabaena socialis*; Xt, *Xenopus tropicalis*; Zm, *Zea mays.* The accession numbers of these enzymes are listed in Supplementary Table S2.

The full-length CDS of *HpWS* (accession OK188764) was obtained *via* RACE. It encodes a 586-AA protein. The HpWS protein is predicted to contain three transmembrane domains near its C-terminus (Supplementary Figure S1). In addition, it is predicted that multiple hydrophobic helixes are located in the first 200 AA in its N-terminus. The phylogenetic analysis indicated that HpWS belongs to the MBOAT superfamily, clustered with acyl CoA:sterol acyltransferase (ASAT) or acyl-CoA wax alcohol acyltransferase (AWAT) from the plant and algae. It is closer to the DGAT1 family than the DGAT2 family (Figure 1A). The WS clade was different from the reported bifunctional WS/DGAT clade. The enzymes in the former clade possess multiple transmembrane domains, and the enzymes in the latter clade are usually soluble or possess only one transmembrane domain (Supplementary Figure S1). The predicted topological structure of HpWS and orthologs also indicated that they are closer to the DGAT1 family with multiple transmembrane domains (Supplementary Figure S1) (Turchetto-Zolet et al., 2011). However, the conserved “FYXDWWN” motif in DGAT1 and ASAT from organisms other than plants was missing in the plant/algae WS clade. Instead, another “RRWNL” and several different conserved motifs were found (Figure 1B). This indicates the distinct origin of this protein clade. Additionally, compared with other enzymes in plant/algae WS clade, the AA sequence of HpWS showed extra 140-AA residues without conserved sequences flanking at C-terminus. The predicted transmembrane domain of HpWS was located in this extra area (Supplementary Figure S1). These results indicate that HpWS belongs to a new scarcely characterized family.

### Bifunction of HpWS

**FIGURE 2:**
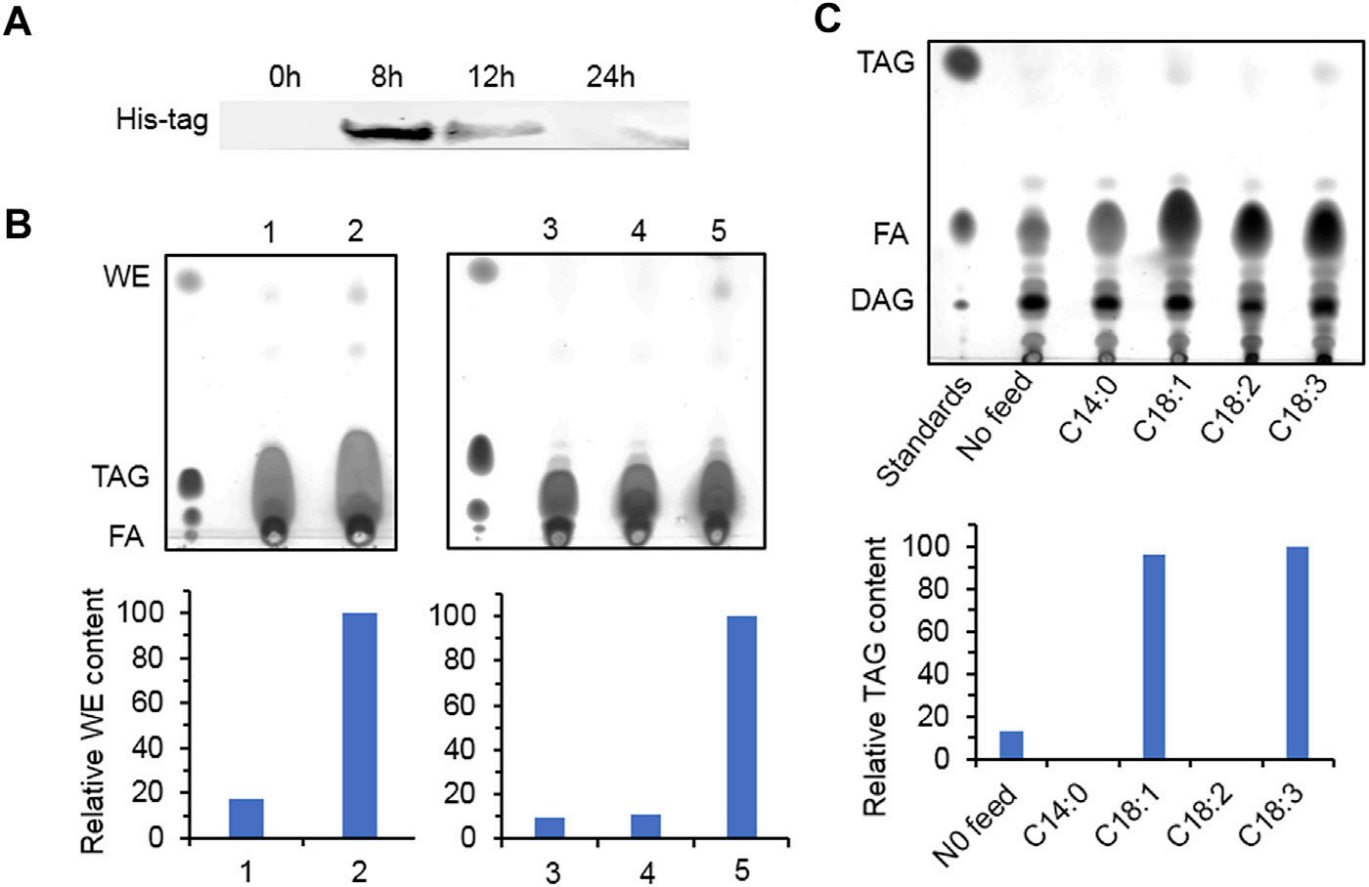
Functional study of HpWS in the *Saccharomyces cerevisiae* H1246 system. **(A)** Expression of recombinant HpWS in yeast microsomes after induction for up to 24 h at 25°C. **(B)** Thin-layer chromatography (TLC) separation of wax esters produced in *S. cerevisiae* harboring empty vector (EV) (1) or *HpWS* (2) fed with 0.1% (*w*/*v*) C16:0 FA + C16 alcohol + C18 alcohol; *S. cerevisiae* harboring *HpWS* fed with 0.1% (*w*/*v*) C16:0 FA (3), C16:0 FA + C16 alcohol (4), or C16:0 FA + C18 alcohol (5). **(C)** TLC separation of TAGs produced in *S. cerevisiae* harboring *HpWS* fed with 0.1% (*w*/*v*) four species of fatty acid. Relative contents of WE and TAG were quantified by ImageJ. FA, fatty acid; WE, wax ester; TAG, triacylglycerol; DAG, diacylglycerol.

Yeast mutant *S. cerevisiae* H1246 contains knockout of *DGA1*, *LRO1*, *ARE1*, and *ARE2* and cannot synthesize neutral lipid including TAG and SE. It is usually employed to study the function of DGAT. After 8 h of induction, the recombinant protein accumulated the most in the transformants overexpressing *HpWS* (Figure 2A). However, the recombinant protein expression decreased with time and almost disappeared at 24 h. For WE production, the control group fed with C16:0 FA and two fatty alcohol species (C16 and C18) produced a trace amount of WEs (Figure 2B, lane 1). The pYES-HpWS-overexpressing yeast fed with the same FA and fatty alcohols accumulated more WEs (Figure 2B, lane 2) than the control group. The relative WE content of the control and the pYES-HpWS was 17 and 100, respectively. When only C16:0 FA was fed, no WEs were produced in the pYES-HpWS-overexpressing yeast (Figure 2B, lane 3). When C16:0 FA and C16 alcohol were fed, no WEs were detected in pYES-HpWS-overexpressing yeast (Figure 2B, lane 4). When C16:0 FA and C18 alcohol (Figure 2B, lane 5) were fed, the WE accumulated in pYES-HpWS-overexpressing yeast, indicating that HpWS functioned as WS and preferred C18 alcohol as the substrate. The relative WE content was in lanes 3, 4, 5 was 9, 11, and 100. In addition, when fed with free FA C18:1 and C18:3, the TAG was synthesized in pYES-HpWS-overexpressing yeast. No TAG was formed in pYES-HpWS-overexpressing yeast fed with C14:0 and C18:2. The relative TAG content in no feed, C14:0, C18:1, C18:2, and C18:3 was 13, 0, 96, 0, and 100, respectively. Feeding with C16:0 also did not re-establish the TAG synthesis in pYES-HpWS-overexpressing yeast (Figure 2B, lanes 3–5). These results indicate that HpWS is a bifunctional WS/DGAT and prefers C18 substrates.

### The Involvement of HpWS in Triacylglycerol Accumulation at Early Stage Under High-Light Stress

**FIGURE 3:**
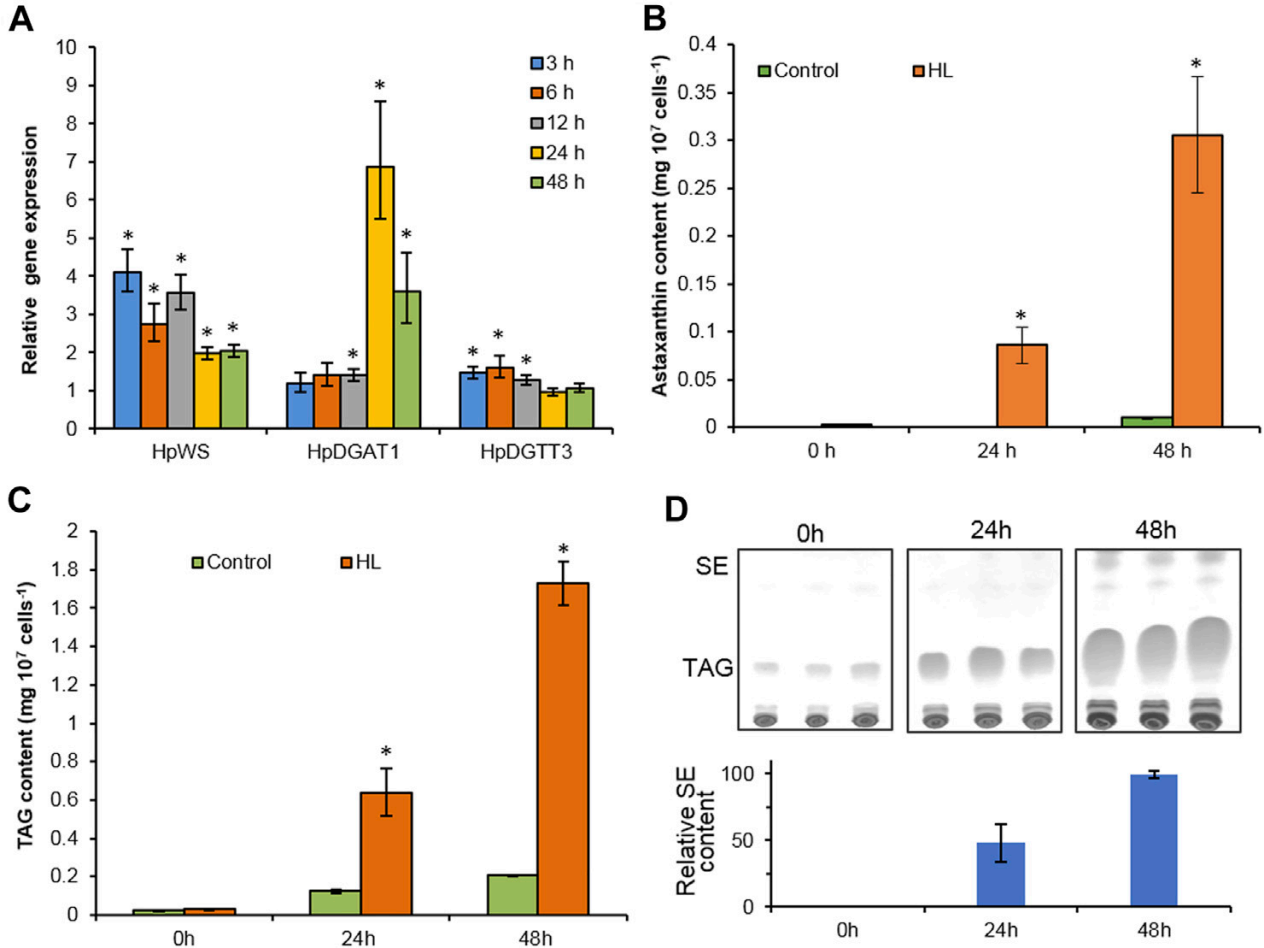
High-light-induced accumulation of neutral lipids in *Haematococcus pluvialis NIES-144*. **(A)** The relative gene expression of *HpWS*, *HpDGAT1*, and *HpDGTT3*. **(B)** Astaxanthin content. **(C)** TAG content. **(D)** Thin-layer chromatography (TLC) separation of sterol esters produced. Control, low light; HL, high light; TAG, Triacylglycerol; SE, sterol ester. Data are expressed as mean ± SD, *n* = 3. *p < 0.05 (Student’s *t*-test). The lipid residue was re-established in chloroform, and the volume was normalized to cell number. An equal volume of each sample was loaded on TLC for separation.

TAGs, astaxanthin esters, and a trace amount of SEs accumulated in *H. pluvialis* under HL stress (Boussiba, 2000; Lee and Zhang, 1999). In order to investigate the possible physiological function of HpWS, we compared the expression of three genes, *HpWS*, *HpDGAT1*, and *HpDGTT3*. The content of both TAGs and astaxanthin esters in HL-stressed *H. pluvialis* changes over time (Figure 3). The expression of *HpDGAT1* was 0.4-, 5.9-, and 2.6-fold significantly upregulated after 12, 24, and 48 h under HL stress, respectively (Figure 3A). However, the expression of *HpWS* responded rapidly to HL stress and was significantly upregulated by 3.1-, 1.7-, 2.6-, 1.0-, and 1.0-fold after 3, 6, 12, 24, and 48 h, respectively. The expression of *HpDGTT3* encoded a confirmed TAG synthase and showed a similar upregulation pattern to that of *HpWS* before 12 h under HL stress with less extent (Figure 3A). Under the control condition, astaxanthin was hardly detected in *H. pluvialis* cells. In contrast, under HL stress, the content of astaxanthin accumulated to 0.08 and 0.31 mg 10^7^ cells^−1^ at 24 and 48 h, respectively (Figure 3B). The accumulation of astaxanthin esters mainly started after 24 h under HL stress, because only 28% of astaxanthin esters were synthesized at 24 h (Figure 3B). Under the control condition, TAG accumulated in *H. pluvialis* cells, and the contents were 0.02, 0.12, and 0.21 mg 10^7^ cells^−1^ at 0, 24, and 48 h, respectively (Figure 3C). The TAG contents in the HL stressed *H. pluvialis* reached 0.03, 0.64, and 1.73 mg 10^7^ cells^−1^ at 0, 24, and 48 h, respectively. They were significantly greater than those in the control group. Nearly 40% of TAG accumulated at 24 h under HL stress. This indicates that the biosynthesis of TAG responded faster than that of astaxanthin esters under HL stress. Previous studies indicated that HpDGAT1 is the major TAG synthase in *H. pluvialis* (Ma et al., 2020). However, *HpDGAT1* was only upregulated after 24 h under HL stress. This indicates that other TAG synthase contributed to TAG accumulation in *H. pluvialis* at an early stage under HL stress. In addition, another group of neutral lipids was produced in HL stressed *H. pluvialis*, and the content of these lipids was less than that of the TAGs (Figure 3D). Through the identification of GC and GC-MS, these lipid species were confirmed to be SEs with gamma. Ergosterol and stigmasta-7,16-dien-3-ol as the dominant sterol moiety instead of WEs (Supplementary Figure S2).

**FIGURE 4:**
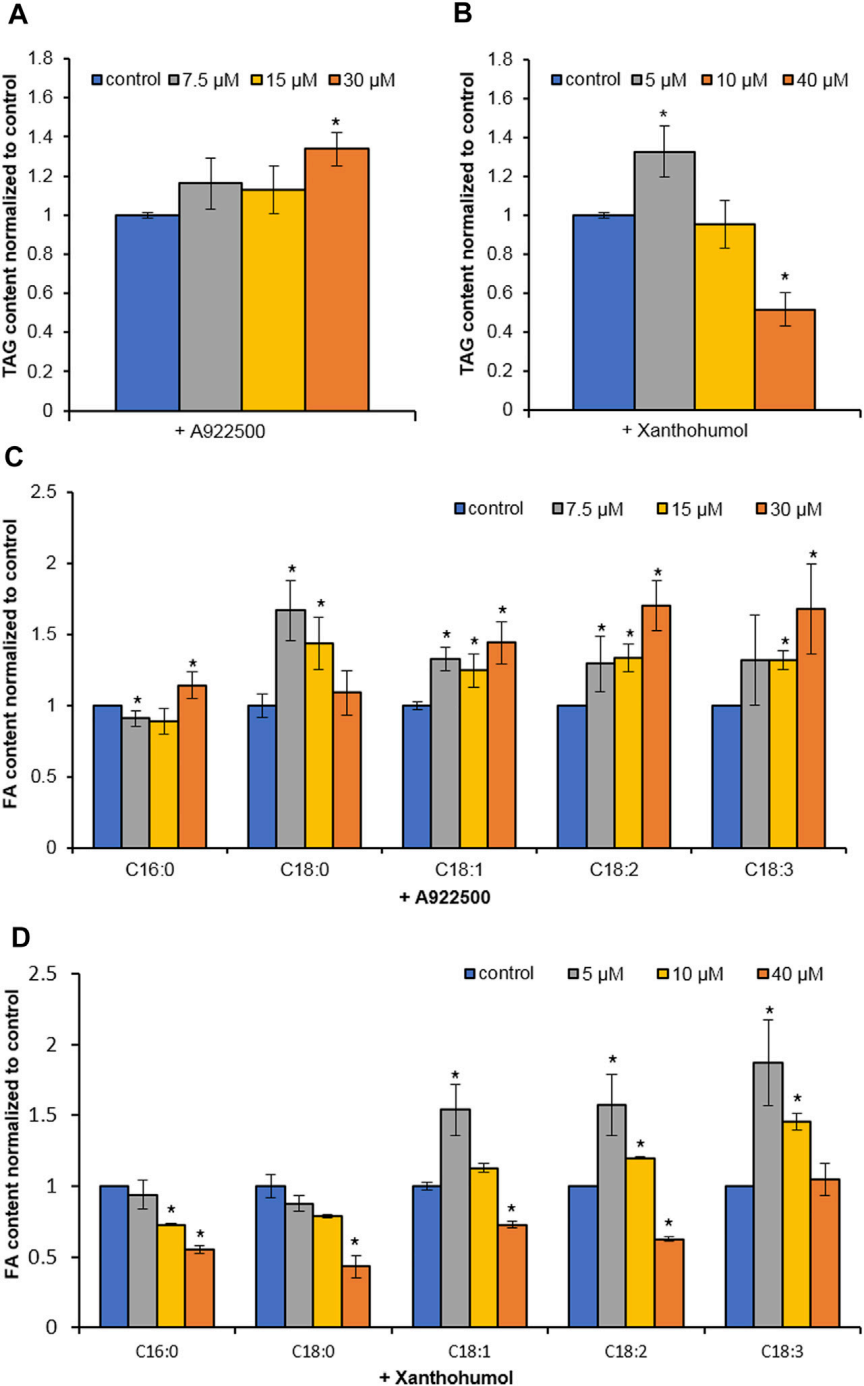
Effect of DGAT inhibitors on TAG biosynthesis in *Haematococcus pluvialis* under HL stress for 24 h. **(A)** TAG content in *H. pluvialis* fed with A922500 ranged from 7.5 to 30 µM. **(B)** TAG content in *H. pluvialis* fed with xanthohumol ranged from 5 to 40 µM. **(C)** Fatty acid content in *H. pluvialis* fed with A922500 ranged from 7.5 to 30 µM. **(D)** Fatty acid content in *H. pluvialis* fed with xanthohumol ranged from 5 to 40 μM. A922500, DGAT1-specific inhibitor; xanthohumol, DGAT1, and DGAT2 inhibitor. Data are expressed as mean ± SD, *n* = 3. *p < 0.05 (Student’s *t*-test).

Two DGAT inhibitors, A922500 and xanthohumol, were introduced to further confirm the role of HpWS in the TAG synthesis in *H. pluvialis* under HL stress. The application of DGAT1-specific inhibitor, A922500, with a concentration of 30 μM did not inhibit the TAG biosynthesis at 24 h under HL stress (Figures 4A,C). The *H. pluvialis* culture applied with 30 μM of A922500 showed a red color similar to the HL group (Supplementary Figure S3). Except for C16:0 of A922500 at a concentration of 7.5 μM, most FA species in TAGs did not decrease (Figure 4C). When cultured with 30 μM of A922500, the TAG content and the FA species in TAGs in *H. pluvialis* increased significantly. These results indicate that HpDGAT1 did not contribute to TAG accumulation at an early stage under HL stress. The relative expressions of *HpWS* and five copies of *HpDGATs* were not significantly influenced in HL + 30 μM of A922500 than those in the HL group except for *HpDGTT3* and *HpDGTT4* (Supplementary Figure S4). The increased expression of *HpDGTT3* might contribute to the enhanced TAG content in the HL + 30 μM of A922500 group. When DGAT1/DGAT2 inhibitors, 5 μM of xanthohumol, were introduced to *H. pluvialis*, the TAG production increased. When 40 μM of xanthohumol was introduced, the TAG production significantly decreased 48% (Figure 4B). The TAG produced at 24 h was 80% (0.12 vs. 0.64 mg 10^7^ cells^−1^) more than that with LL (control1) (Figure 3C). However, when treated with 40 μM of xanthohumol, the TAG content was *ca*. 50% less than that with HL (control2) (Figure 4B). These data indicated that 30% (80%–50%) TAG was still produced under HL + 40 μM of xanthohumol. The relative expressions of *HpWS*, *HpDGAT1*, *HpDGTT2*, and *HpDGTT3* in HL + 40 μM of xanthohumol were not significantly different from those in the HL group (Supplementary Figure S4). However, the relative expressions of *HpDGTT1* and *HpDGTT4* were significantly decreased. Consequently, we proposed that TAG synthase other than DGAT was involved in TAG production at the early stage of HL stress. The *H. pluvialis* culture with 40 μM of xanthohumol was almost bleached under HL stress (Supplementary Figure S3). This indicates that DGAT1 and DGAT2 are crucial for *H. pluvialis* survival under HL stress. With the presence of 5 μM of xanthohumol, the contents of C18:1, C18:2, and C18:3 were significantly increased (Figure 4D). With 40 μM of xanthohumol, only the content of C18:3 was not significantly different from that of the control group. This indicates that this FA species was incorporated into TAGs by acyltransferase other than DGAT1 or DGAT2. Overall, these data indicated that HpWS was involved in TAG biosynthesis in *H. pluvialis* at the early stage under HL stress.

### Potential Application of HpWS in Biotechnology

**FIGURE 5:**
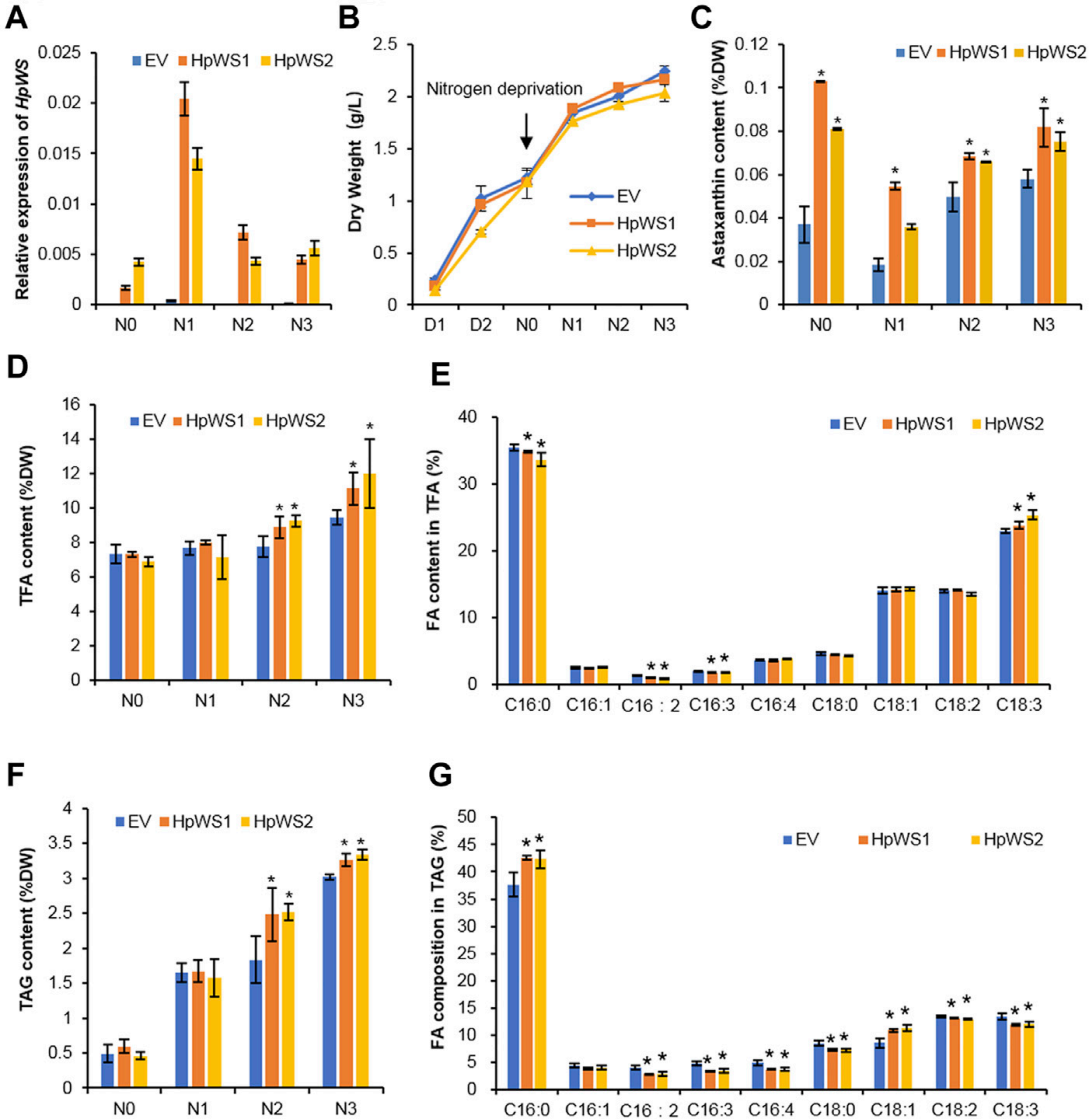
Growth and lipid production in *Chlamydomonas reinhardtii* BKT5 transformants overexpressing HpWS under nitrogen-replete and nitrogen-depleted conditions. **(A)** Expression of *HpWS* in the *C. reinhardtii* transformants. **(B)** Dry weight. **(C)** Astaxanthin content. **(D)** TFA content. **(E)** Fatty acid composition in TFA after 3 days under nitrogen deprivation. **(F)** TAG content. **(G)** Fatty acid composition in TAG after 2 days under nitrogen deprivation. TFA, total fatty acid; FA, fatty acid. EV, *C. reinhardtii* BKT5 harboring pChamy_4 vector. HpWS1 and HpWS2 are two individual *C. reinhardtii* transformants overexpressing HpWS. D, culture under nitrogen repletion; N, culture under nitrogen deprivation. Data are expressed as mean ± SD, *n* = 4. *p < 0.05 (Student’s *t*-test).

To clarify HpWS functions as astaxanthin acyltransferase, HpWS was overexpressed in *C. reinhardtii bkt5*, which produces free astaxanthin (Perozeni et al., 2020). Two *C. reinhardtii bkt5* transformants, HpWS1 and HpWS2, with relatively high expression of *HpWS* were screened out (Figure 5A). The relative expressions of six copies of *CrDGAT* were also determined in these *HpWS*-overexpressing strains (Supplementary Figure S5). *CrDGAT1*, *CrDGTT1*, *CrDGTT2*, and *CrDGTT3* showed upregulation upon nitrogen-deprivation stress, suggesting that these DGATs might be involved in TAG biosynthesis in *C. reinhardtii bkt5*. In some cases, the relative expressions of some *CrDGATs*, such as *CrDGTT1*, *CrDGTT2*, and *CrDGTT3* in HpWS1 and HpWS2, were significantly lower than those in the EV at day 3 after nitrogen-deprivation stress. These data indicated that the activity of some of the endogenous DGATs might be weakened in these *HpWS*-overexpressing strains. There was no significant difference in the dry weight accumulation among the EV (empty vector transformant) and two transformants overexpressing HpWS (Figure 5B). The dry weight of the three strains reached about 1.2 g L^−1^ after 3 days of cultivation under nitrogen-replete conditions and continued to increase to about 2.0 g L^−1^ after another 3 days of cultivation under nitrogen-depleted conditions. Although neither astaxanthin esters nor other xanthophyll esters were detected in the HpWS1 and HpWS2, the content of free astaxanthin was substantially enhanced in the two strains (Figure 5C). Under nitrogen repletion, the astaxanthin contents of EV, HpWS1, and HpWS2 in Dry Weight (DW) were 0.04%, 0.10%, and 0.08%, respectively. This indicates that the astaxanthin production was up to 1.5-fold promoted in the HpWS-overexpressing strains. Under nitrogen-deficient conditions, compared with a nitrogen-sufficient condition, the astaxanthin content in EV, HpWS1, and HpWS2 under nitrogen-deficient conditions on day 1 was 50% lesser than that under nitrogen-sufficient conditions. In addition, the astaxanthin contents of the three strains in DW on day 3 were gradually increased. They reached 0.06%, 0.08%, and 0.07%, respectively. Compared with the case of nitrogen-sufficient condition, only the astaxanthin content in EV was increased after 3 days under nitrogen-deficient conditions. The astaxanthin content in HpWS-overexpressing strains under nitrogen-deficient conditions was lower than that under nitrogen-sufficient conditions.

The contents of total FA (TFA) and TAG in the transformants overexpressing HpWS under nitrogen-deficient conditions were significantly increased (Figures 5D,F). The TFA content in EV was 7.3% of that in DW under nitrogen-replete conditions. It was gradually increased to 9.4% of that in DW on day 3 under nitrogen-depleted conditions. In HpWS1 and HpWS2, the contents of TFA were increased by 15.6% and 19.5% on day 2 and day 3. They were 18.1% and 27.7% more than that of EV under nitrogen deprivation, respectively (Figure 5D). Likewise, the contents of TAG in HpWS1 and HpWS2 were 39% and 11% enhanced on day 2 and day 3 under nitrogen-depleted conditions, respectively (Figure 5F). Although the expression of some endogenous DGATs was weakened in HpWS1 and HpWS2 (Supplementary Figure S5), the contents of both TAGs and TFA were significantly increased in these strains than those in the EV. This indicated the strong TAG synthase activity of HpWS in the heterologous host, since the contents of TFA and TAGs increased the most on day 3 and day 2 under nitrogen-depleted conditions, respectively. The compositions of FAs in the correspondent samples were investigated (Figures 5E,G). Among the nine FA species detected in TFA, the proportions of C16:0, C16:2, and C16:3 were significantly reduced in the HpWS-overexpressing transformants, and the proportion of C18:3 was significantly increased (Figure 5E). For the FA composition in TAGs, the proportions of all the FA species in the HpWS-overexpressing transformants significantly changed except for C16:1 (Figure 5G). Only the proportions of C16:0 and C18:1 in HpWS1 and HpWS2 were significantly increased. These results indicate that overexpressing HpWS in *C. reinhardtii bkt5* significantly increased the proportions of C18:3 and C18:1 in TFA and TAGs, respectively. This was consistent with the predicted substrate preference in yeast *S. cerevisiae* H1246 system (Figure 2). No WE was detected in the HpWS-overexpressing *C. reinhardtii bkt5* strains under both nitrogen-replete and nitrogen-depleted conditions (Supplementary Figure S6). This indicates that HpWS did not produce WEs in *C. reinhardtii* either.

## Discussion

In microalgae and plants, TAGs are mainly synthesized by the acyl-CoA-dependent Kennedy pathway. The final acylation accomplished by DGAT is the key and rate-limiting step. There are three families of DGAT, including two membrane-bound families of DGAT1 and DGAT2, along with a cytosolic DGAT3 (Turchetto-Zolet et al., 2011; Chi et al., 2014). DGAT1 and DGAT2 have non-redundant functions in TAG biosynthesis in terms of localization, expression pattern to environmental stress or signals during development, substrate preferences, and so on (Shockey et al., 2006; Boyle et al., 2012; Wei et al., 2017; Mao et al., 2019; Ma et al., 2020).

In addition to these canonic DGATs, phospholipid DAG acyltransferase (PDAT) was found to contribute to TAG accumulation by degrading membrane lipids at the early stages of nitrogen deprivation (Yoon et al., 2012). In a previous study, the orthologs of CrPDAT in *H. pluvialis* did not show upregulation of expression (Ma et al., 2020). This indicates that PDAT was not involved in TAG accumulation in *H. pluvialis* under HL stress. On the contrary, HpWS promoted TAG accumulation at the early stages under HL stress through the acyl-CoA dependent pathway. In addition to HpWS, there are several other types of acyltransferases with multiple functions, such as WSD1, PES1, and PES2 from *A. thaliana* (Li et al., 2008; Lippold et al., 2012); CzWS1 from *Ch. zofingiensis* (Xu et al., 2021); and PtWS from *P. tricornutum* (Cui et al., 2018). The TAG synthase activity of these multifunctional acyltransferases suggests that they may also be involved in TAG production during abiotic stresses or physiological processes such as senescence in plants or microalgae.

HpWS characterized in this study belongs to a novel plant/algae WS family. One of the orthologs was characterized in microalga *Ch. zofingiensis* (Xu et al., 2021). Like *H. pluvialis*, *Ch. zofingiensis* has no detectable WEs. However, the transcript of *CzWS1* was highly abundant under stress (Xu et al., 2021). This brought the mysterious physiological function of this gene family in microalgae. Some microalgal species, such as *E. gracilis*, produce WEs when the culture condition changes, including those from aerobic to anaerobic conditions or from light to dark (TUCCI et al., 2010). The WE from *E. gracilis* was EgWS, which belongs to the same plant/algae WS clade as HpWS and CzWS1. This protein family was closer to the DGAT1 superfamily (Figure 1). This indicates that these plant/algae WS orthologs evolved from the same ancestor with DGAT1s. In *P. tricornutum*, the wild-type strain did not produce WEs unless PtWS/DGAT was overexpressed (Cui et al., 2018). Probably, in *H. pluvialis* and *Ch. Zofingiensis*, the induction condition remains to be explored because of the lack of precursor, such as long-chain fatty alcohol in these species under unfavorable conditions, such as HL or nitrogen deprivation. The appropriate induction conditions in *E. gracilis*, such as from light to dark or aerobic to anaerobic conditions, are worthy of further exploration in the future.

A number of acyltransferases identified from plants and microalgae meet the demand of biotechnological application in enhancing TAG production through endogenous or heterologous overexpression (Cui et al., 2018; Maravi et al., 2016; Yamaoka et al., 2016). Furthermore, some acyltransferases with substrate preferences can change the FA composition in lipid and therefore possess great potential in designing desired lipid species (van Erp et al., 2019; Xu et al., 2021). Likewise, expressing HpWS in *C. reinhardtii bkt5* significantly promoted the accumulation of TFA and TAGs up to 28% and 39%, respectively, without disturbing the biomass accumulation. In addition, the substrate preference of HpWS to C18:1 and C18:3 CoA correspondingly changed the FA compositions of both TFA and TAGs in the *C. reinhardtii bkt5* overexpressing HpWS (Figure 5). The inconsistent modification of FA profile in TFA and TAGs may result from the unidentified activity of HpWS in addition to TAG and WE synthases. PES1 and PES2 from *Arabidopsis* are acyltransferases with multiple functions, which can synthesize SEs, TAGs, and phytol esters (Lippold et al., 2012). Probably, HpWS also incorporated C18 substrates to lipid species other than TAGs and WEs. Furthermore, because C18:1 and C18:3 are the precursors for valuable polyunsaturated FAs, the trait of C18:1/C18:3 preference of HpWS may be applicable in enhancing the eicosapentaenoic acid (EPA) production in microalgal species, such as *Nannochloropsis* and *P. tricornutum* (Scott et al., 2007). In addition, under nitrogen-replete conditions, the production of astaxanthin in HpWS-overexpressing strains was promoted up to 1-fold. HpWS did not act as astaxanthin acyltransferase. The reason is that neither astaxanthin esters nor other xanthophyll esters were detected in *C. reinhardtii bkt5* overexpressing HpWS, according to HPLC chromatogram (not shown). Probably, the overexpression of HpWS redirected the distribution of carbon flux to terpenoid biosynthesis. The underlying mechanism of HpWS promoting the production of free astaxanthin remains to be explored.

In conclusion, our results indicate that HpWS is a plant/algae WS involved in TAG accumulation in *H. pluvialis* at the early stage under HL stress. The expression of *HpWS* in astaxanthin-producing *Chlamydomonas* enhanced the production of oils and astaxanthin. Overall, the findings in this study broaden the understanding of TAG accumulation in microalgae under stress and provide a potential molecular tool for biotechnological application.

## Data Availability

The datasets presented in this study can be found in online repositories. The names of the repository/repositories and accession number(s) can be found below: https://www.ncbi.nlm.nih.gov/, OK188764.
