# Supplementary material for: A Novel Bifunctional Wax Ester Synthase Involved in Early Triacylglycerol Accumulation in Unicellular Green Microalga Haematococcus pluvialis Under High Light Stress
Source: Front Bioeng Biotechnol. 2022 Jan 17;9:794714. doi: 10.3389/fbioe.2021.794714 (PMC8802113; doi:10.3389/fbioe.2021.794714)
Supplement: Supplementary file 1 [file DataSheet2.docx]

**
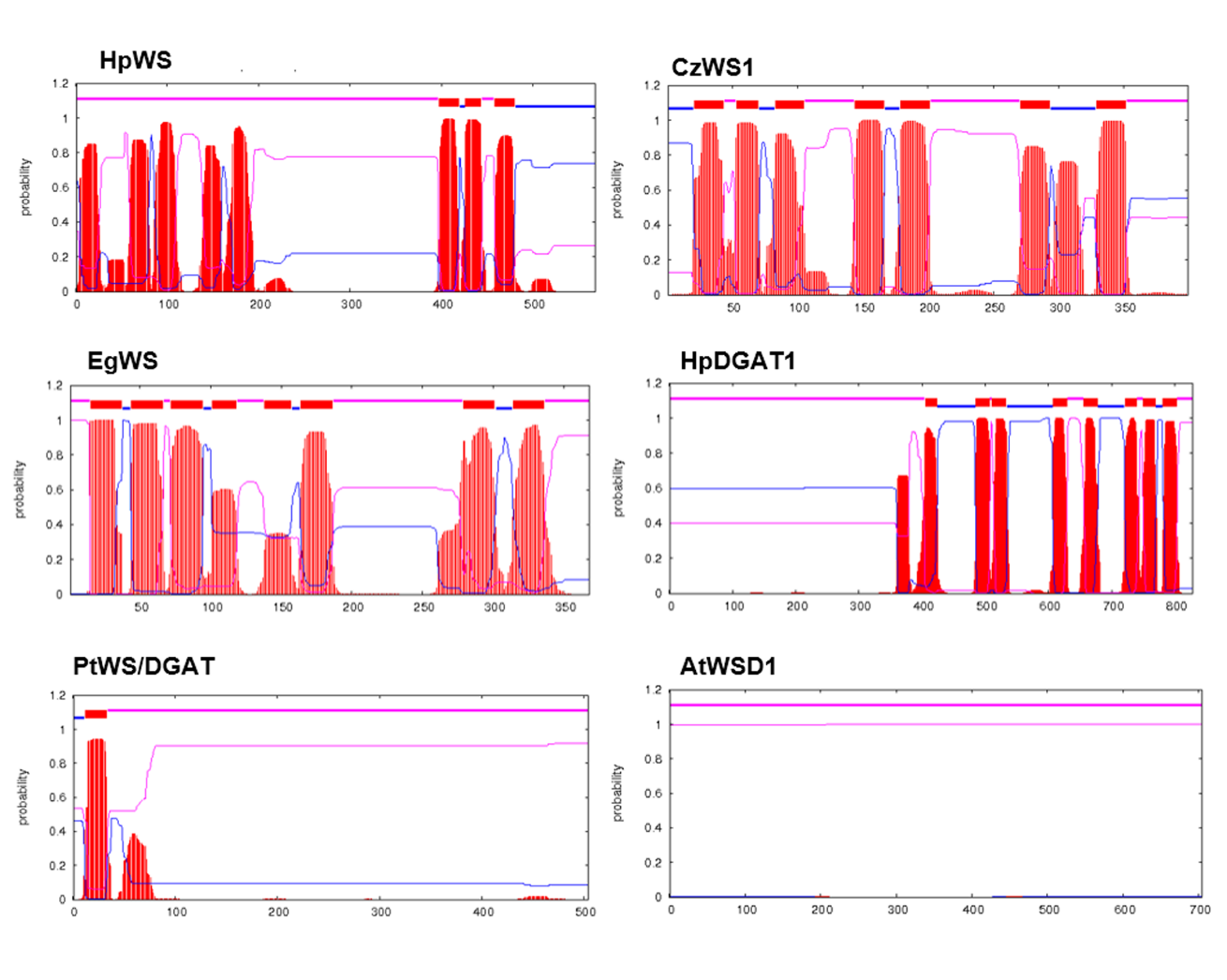
**

**Supplementary Fig. S1 Predicted transmembrane domains of HpWS, CzWS1, EgWS, HpDGAT1, PtWS/DGAT and AtWSD1.** At, *Arabidopsis thaliana* (NP 568547.1); Cz, *Chromochloris zofingiensis* (Cz02g29020); Eg, *Euglena gracilis* (BAV82980.1); Hp, *Haematococcus pluvialis* (WS, UCJ19305.1; DGAT1, MN561784); Pt, *Phaeodactylum tricornutum* (XP 002184474.1).


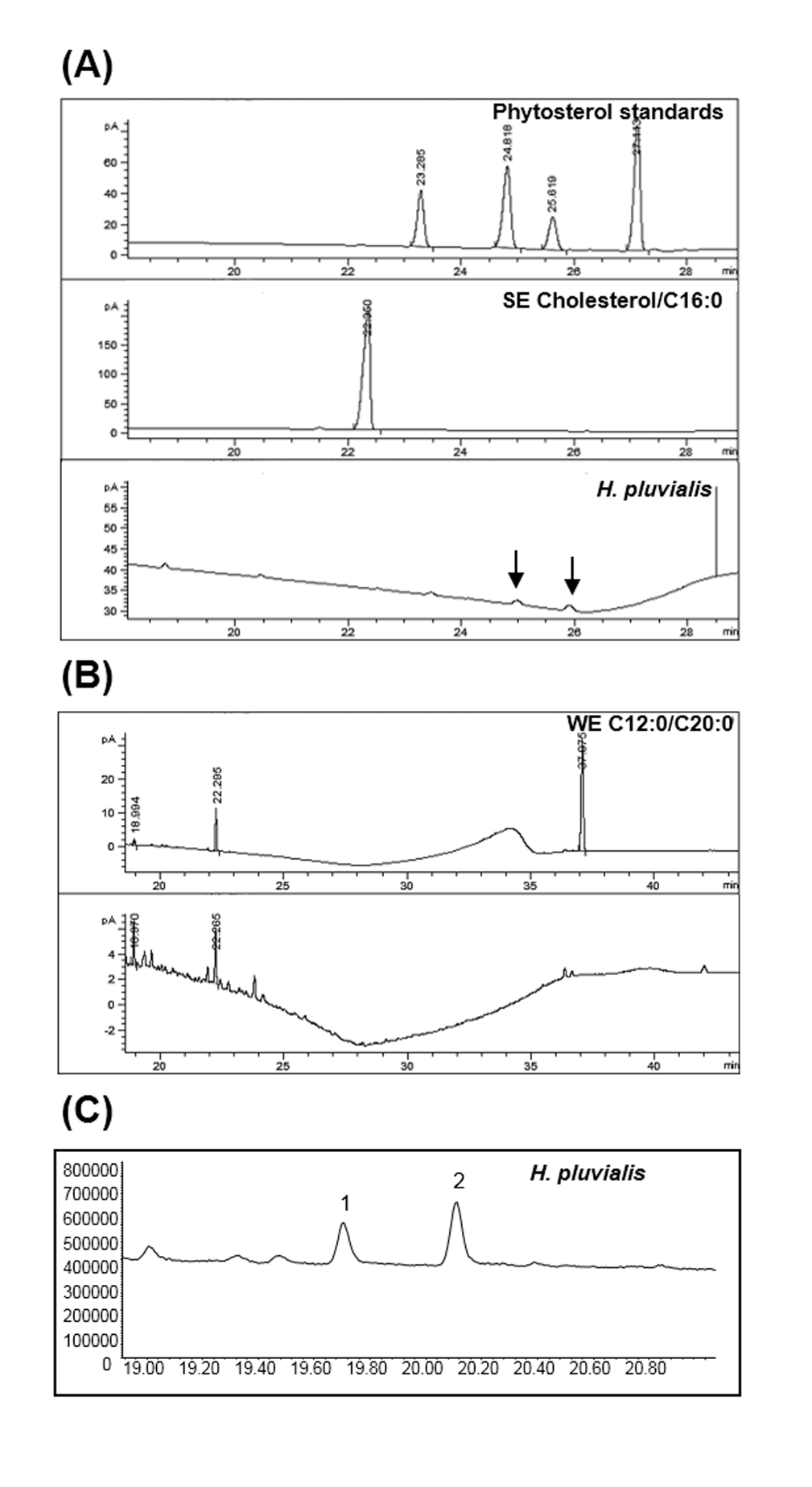


**Supplementary Fig. S2 Identification of sterol esters (SE) produced in *H. pluvialis* under high light stress for 48h.** (A) Detection of sterols species in SE by GC. (B) Detection of wax ester (WE) by GC. (C) Identification of sterol species in SE by GC/MS. Sterol species in *H. pluvialis*: peak 1. .gamma.-Ergostenol; peak 2. Stigmasta-7,16-dien-3-ol, (3.beta.,5.alpha.).


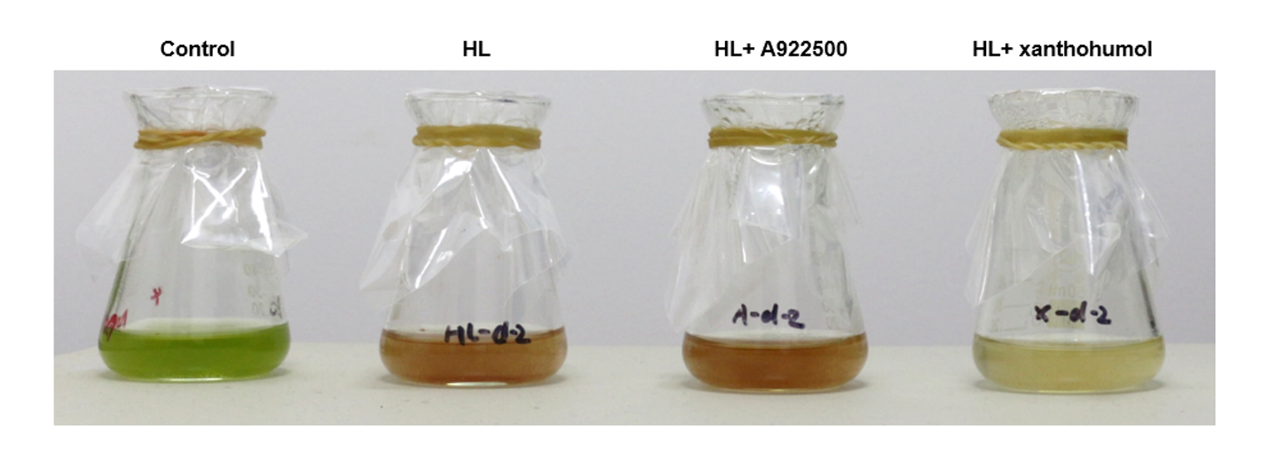


**Supplementary Fig. S3** *H. pluvialis* cultures with or without DGAT inhibitors under high light stressed for 24h. The concentration of A922500 and xanthohumol was 30 μM and 40 μM, respectively.


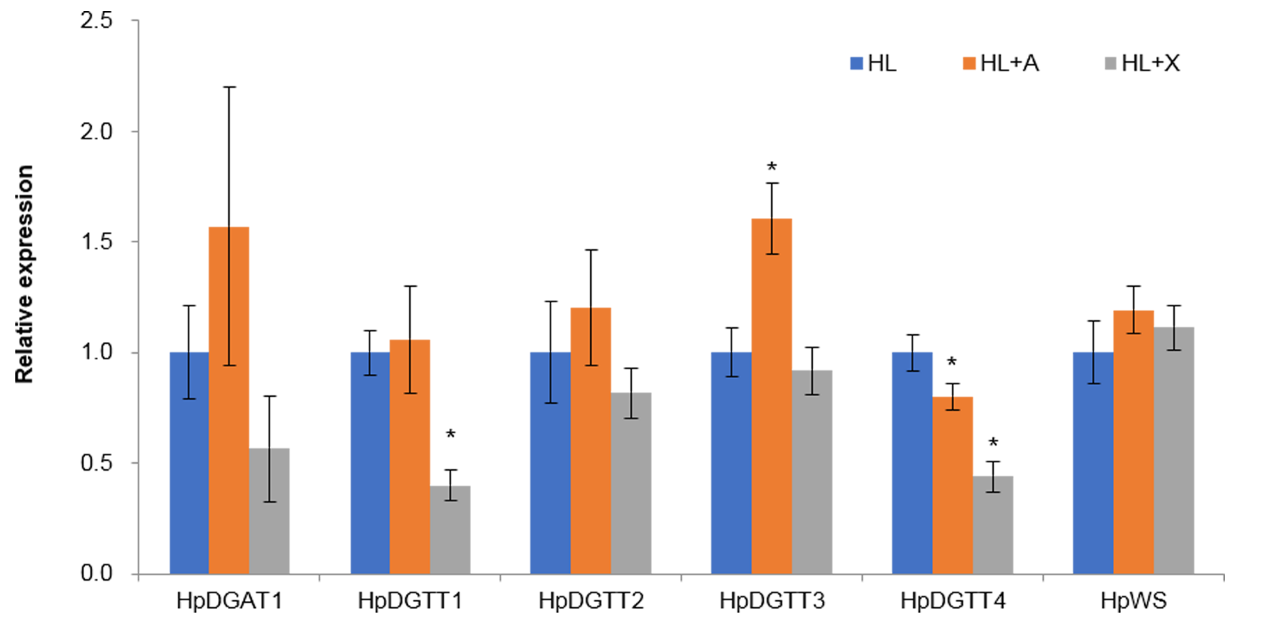


**Supplementary Fig. S4** Relative expression of HpWS and five copies of HpDGATs under high light stress for 24 h with application of 30 μM A922500 or 40 μM xanthohumol. Data are expressed as mean ± SD, n=3. *P<0.05 (Student’s t-test).  HL, high light; HL+A, high light+30 μM A922500; HL+X, HL+40 uM xanthohumol.


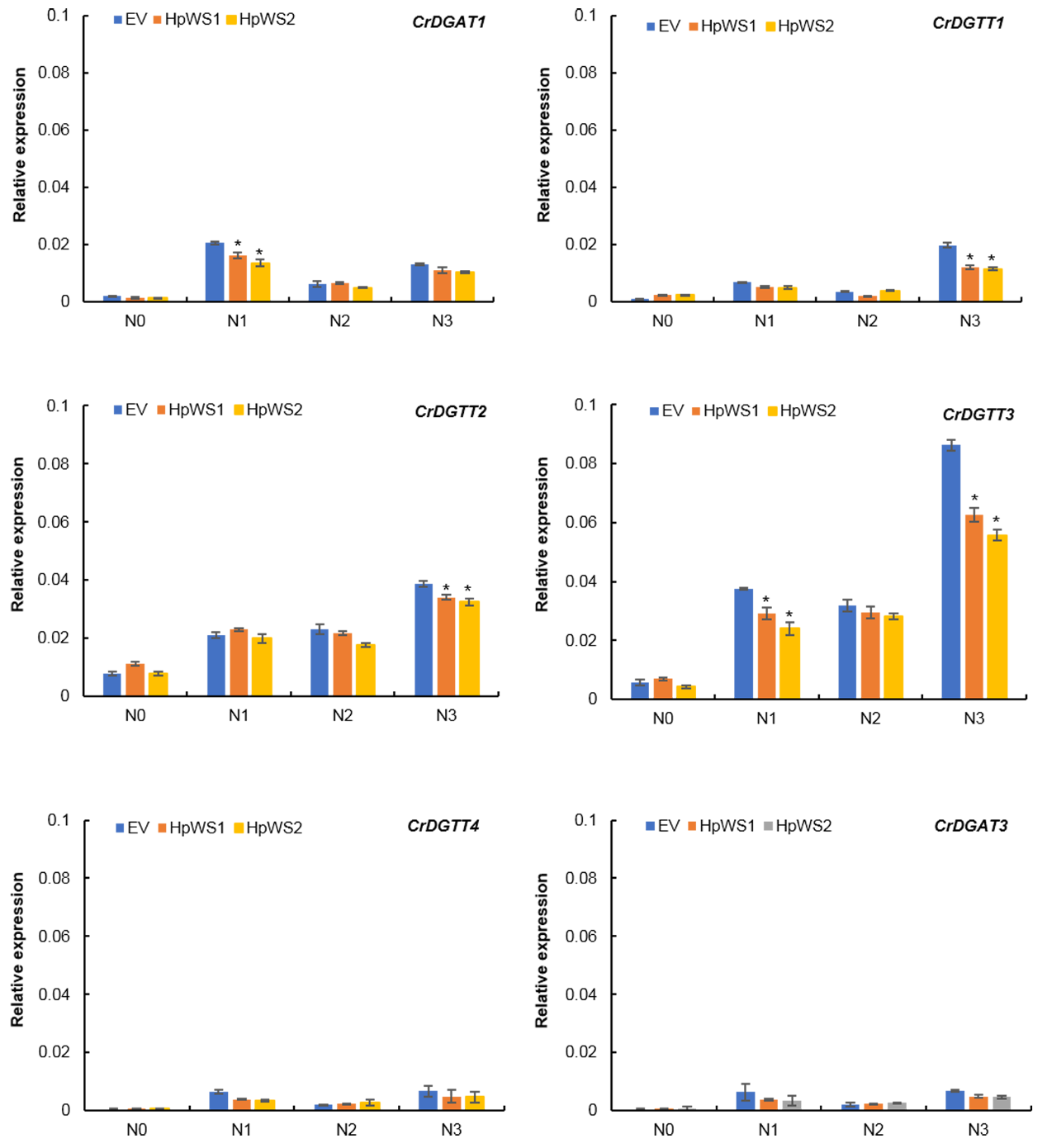


**Supplementary Fig. S5** Relative expression of six copies of *CrDGATs* in the *HpWS* overexpressing transformants. Data are expressed as mean ± SD, n=4. *P<0.05 (Student’s *t*-test).  N, culture under nitrogen deprivation.


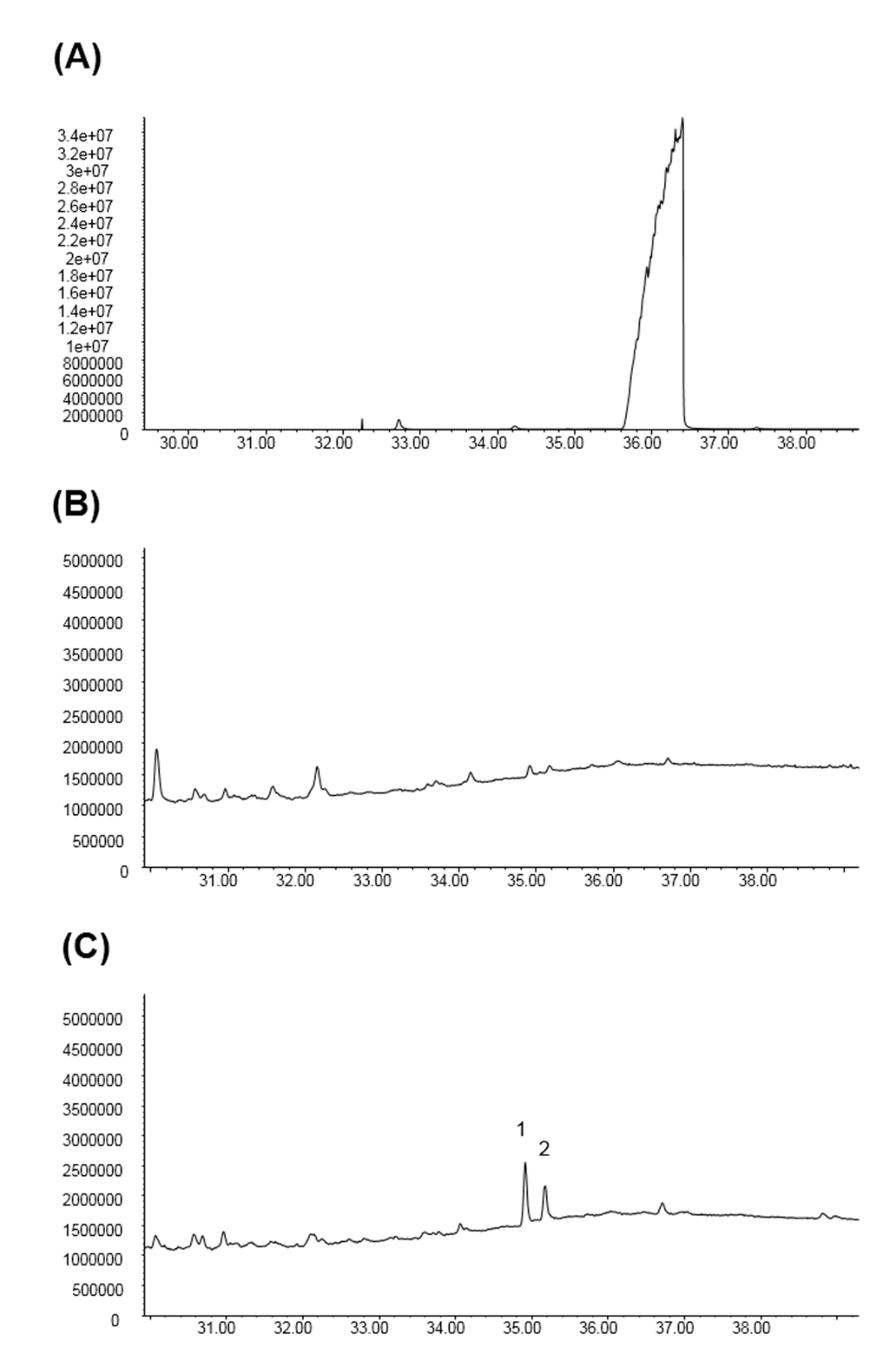


**Supplementary Fig. S6 Detection of wax esters (WE) produced in HpWS overexpressing *C. reinhardtii* bkt5 transformants by GC-MS.** (A) Chromatogram of WE C12:0/C20:0 standard. (B) Chromatogram of neutral lipids in HpWS1 and HpWS2 mix under nitrogen replete condition. (C) Chromatogram of neutral lipids in HpWS1 and HpWS2 mix under nitrogen deplete condition for 3 days. Peak 1 and peak 2 were free sterols, which were identified to be Stigmast-7-en-3-ol, (3.beta.,5.alpha.,24S)- and fucosterol, respectively.
