# Supplementary material for: A Novel Bifunctional Wax Ester Synthase Involved in Early Triacylglycerol Accumulation in Unicellular Green Microalga Haematococcus pluvialis Under High Light Stress
Source: Front Bioeng Biotechnol. 2022 Jan 17;9:794714. doi: 10.3389/fbioe.2021.794714 (PMC8802113; doi:10.3389/fbioe.2021.794714)
Supplement: Supplementary file 2 [file DataSheet1.docx]

**Supplementary Table S1** Primers list

| **Application** | **Name** | **Sequence** |
| --- | --- | --- |
| RACE | SmFs | GCAGTGGTATCAACGCAGAGTGGCCAT |
|  | HpWS-R1 | CCGACGCTGGAACCTAAGCACCACCTA |
|  | HpWS-n-R2 | GGCCCCAGCTTTCAACAGGCCATGGAT |
|  | HpWS-n-R3 | CCCGACGCTGGAACCTAAGCACCACCT |
|  | SmRs | TTCTAGAGGCCGAGGCGGCCGACAT |
|  | HpWS-F1 | GCGTCGGGCCCAGAAGTCAGCA |
|  | HpWS-n-F2 | TCCAGCGTCGGGCCCAGAAGTCA |
|  | HpWS-n-F3 | GCGTCGGGCCCAGAAGTCAGCAA |
| Constructing pYES2-HpWS and pChlamy-HpWS vector | HpWax-F-EcoRI | CCGgaattcATGATTGAGCTGCT |
|  | HpWax-R-XhoI | CCGctcgagCACTGCATGGATTGCT |
| Gene expression in *Haematococcus pluvialis* under high light stress | 18s rRNA-F | TGCCTAGTAAGCGCGAGTCA |
|  | 18s rRNA-R | CCCACCGCTAAAGTCAATCC |
|  | HpDGAT1-qF | CTGCTGCGCCACGTCTACTT |
|  | HpDGAT1-qR | GTTGCCAGCTTCGTCCCTGT |
|  | HpDGTT1-qF | TGTCACAACCCGGGACATGC |
|  | HpDGTT1-qR | CCGTGAATGAGGGGGATGGC |
|  | HpDGTT2-qF | ACGCAAGGGGTTTGTGGAGG |
|  | HpDGTT2-qR | GGGGATAGGTGACCCCGTGA |
|  | HpDGTT3-qF | GAGCAGGTGGACGGCATTGT |
|  | HpDGTT3-qR | GCGCTCAAACTCAGGGTCGT |
|  | HpDGTT4-qF | CAGCTGTGGTGGTGGCTAGG |
|  | HpDGTT4-qR | GGGGCGAAACCAGCTGTAGG |
|  | HpWS-qF | CCAATGCCGCTCTGATTGC |
|  | HpWS-qR | GCCCAGTGCCGTATCAAGT |
| Gene expression in *C. reinhardtii* BKT5 overexpressing HpWS | α-tubulin qF | CTCGCTTCGCTTTGACGGTG |
|  | α-tubulin qR | CGTGGTACGCCTTCTCGGC |
|  | HpWS-qF | CCAATGCCGCTCTGATTGC |
|  | HpWS-qR | GCCCAGTGCCGTATCAAGT |
|  | CrDGAT1-qF | CGGCCGCTGCACTACTCTAT |
|  | CrDGAT1-qR | CGCAACGCGTCATGTTCTC |
|  | CrDGTT1-qF | CTCTGCTCATCGGCACATTG |
|  | CrDGTT1-qR | ATATGCCACTTGCGGAAGGT |
|  | CrDGTT2-qF | CACCGACAAATGTGCGAATT |
|  | CrDGTT2-qR | CACATGCATCCAGCCACAGT |
|  | CrDGTT2-qF | ACCTCGCACTTGACCCTGAA |
|  | CrDGTT2-qR | TCATGAAGCCTACATAAATCGACATC |
|  | CrDGTT2-qF | CGAGCCCCTCACCATTGT |
|  | CrDGTT2-qR | CTGCAGGTCGTCCGTGAAG |
|  | CrDGAT3-qF | GAAATGCTCGGCTCGTTCTC |
|  | CrDGAT3-qR | TCGACCAGCGCGTGTGT |

**Supplemental Table. S2** Information of sequences used for the phylogenetic analysis with HpWS

| **Species name** | **Acronym** | **Accession NO** |
| --- | --- | --- |
| *Acinetobacter baylyi* | AbWS/DGAT | AAO17391.1 |
| *Apis cerana* | AcDGAT1 | [XP_016909650](https://www.ncbi.nlm.nih.gov/protein/XP_016909650?report=genbank&log$=taxrep&RID=CPE3WA6R014) |
| *Arabidopsis thaliana* | AtDGAT1 | [NP_179535](https://www.ncbi.nlm.nih.gov/protein/NP_179535?report=genbank&log$=taxrep&RID=CPE3WA6R014) |
|  | AtMBOAT | NP 200347.1 |
|  | AtWSD1 | NP 568547.1 |
|  | AtPES1 | AAO64893.1 |
|  | AtPES2 | Q9LW26.1 |
| *Aspergillus niger* | AnDGAT2A | [RDH24132](https://www.ncbi.nlm.nih.gov/protein/RDH24132?report=genbank&log$=taxrep&RID=CPFF15Y7014) |
| *Brassica napus* | BnDGAT1 | [NP_001303201](https://www.ncbi.nlm.nih.gov/protein/NP_001303201?report=genbank&log$=taxrep&RID=CPE3WA6R014) |
|  | BnASAT | XP 013698964.1 |
|  | BnAWAT | XP 013646834.1 |
|  | BnDGAT2 | [XP_013745595](https://www.ncbi.nlm.nih.gov/protein/XP_013745595?report=genbank&log$=taxrep&RID=CPFP7ZTD016) |
|  | BnWSD1 | XP 013687456.1 |
|  | BnAcyltransferase | XP 013707442.1 |
| *Brassica rapa* | BrAcyltransferase | XP 009113427.1 |
| *Capsella rubella* | CarAcyltransferase1 | XP 006300403.1 |
|  | CarAcyltransferase2 | XP 006290672.1 |
| *Chlamydomonas eustigma* | CeDGAT1 | [GAX78660](https://www.ncbi.nlm.nih.gov/protein/GAX78660?report=genbank&log$=taxrep&RID=CPE3WA6R014) |
| *Citrus sinensis* | CisAcyltransferase | XP 024951659.1 |
| *Coffea eugenioides* | Coe | XP 027183708.1 |
| *Chlamydomonas reinhardtii* | CrDGTT1 | [AGO32156](https://www.ncbi.nlm.nih.gov/protein/AGO32156?report=genbank&log$=taxrep&RID=CPFF15Y7014) |
|  | CrDGTT2 | [AGO32157](https://www.ncbi.nlm.nih.gov/protein/AGO32157?report=genbank&log$=taxrep&RID=CPFJFVVV014) |
|  | CrDGTT3 | [AGO32158](https://www.ncbi.nlm.nih.gov/protein/AGO32158?report=genbank&log$=taxrep&RID=CPFJFVVV014) |
|  | CrDGTT4 | [AGO32159](https://www.ncbi.nlm.nih.gov/protein/AGO32159?report=genbank&log$=taxrep&RID=CPFU5HS7014) |
|  | CrWS (predicted) | XP 001699836.1 |
| *Chlorella sorokiniana* | CsASAT | PRW45129.1 |
|  | CsAWAT | PRW59291.1 |
|  | CsDGAT2A | [PRW21030](https://www.ncbi.nlm.nih.gov/protein/PRW21030?report=genbank&log$=taxrep&RID=CPFU5HS7014) |
|  | CsDGAT2B | [PRW45193](https://www.ncbi.nlm.nih.gov/protein/PRW45193?report=genbank&log$=taxrep&RID=CPFF15Y7014) |
| *Chlorella vulgaris* | CvDGAT1 | [ALP13863](https://www.ncbi.nlm.nih.gov/protein/ALP13863?report=genbank&log$=taxrep&RID=CPE3WA6R014) |
| *Chromochloris zofingiensis* | CzWS1 | Cz02g29020 |
| *Dictyostelium discoideum* | DdDGAT1 | XP_645633.2 |
| *Drosophila melanogaster* | DmDGAT1 | [AAL78365](https://www.ncbi.nlm.nih.gov/protein/AAL78365?report=genbank&log$=taxrep&RID=CPE3WA6R014) |
| *Euglena gracilis* | EgWS | BAV82980.1 |
| *Eutrema salsugineum* | EsAcyltransferase | XP 006395516.1 |
| *Haematococcus pluvialis* | HpDGAT1 | MN561784 |
|  | HpDGTT1 | MN561785 |
|  | HpDGTT2 | MN561786 |
|  | HpDGTT3 | MN561787 |
|  | HpDGTT4 | MN561788 |
|  | HpWS | UCJ19305.1 |
| *Leptolyngbya valderiana* | LvWS/DGAT | WP 063716931.1 |
| *Mus musculus* | MmDGAT1 | [NP_034176](https://www.ncbi.nlm.nih.gov/protein/NP_034176?report=genbank&log$=taxrep&RID=CPE3WA6R014) |
| *Mycobacterium tuberculosis* | MtWS/DGAT | WP078441965.1 |
| *Nannochloropsis oceanica* | NoDGAT1A | [ASL69957](https://www.ncbi.nlm.nih.gov/protein/ASL69957?report=genbank&log$=taxrep&RID=CPE3WA6R014) |
|  | NoDGAT1B | [ASL69958](https://www.ncbi.nlm.nih.gov/protein/ASL69958?report=genbank&log$=taxrep&RID=CPE3WA6R014) |
| *Phaeodactylum tricornutum* | PtWS/DGAT | XP 002184474.1 |
| *Raphanus sativus* | RsAcyltransferase | XP 018434309.1 |
| *Saccharomyces cerevisiae* | ScARE | [AJT04763](https://www.ncbi.nlm.nih.gov/protein/AJT04763?report=genbank&log$=taxrep&RID=CPE3WA6R014) |
| *Tetrabaena socialis* | TsAWAT | PNH01863.1 |
|  | TsDGAT2 | [PNH06113](https://www.ncbi.nlm.nih.gov/protein/PNH06113?report=genbank&log$=taxrep&RID=CPFJFVVV014) |
|  | TsDGAT2A | [PNH04213](https://www.ncbi.nlm.nih.gov/protein/PNH04213?report=genbank&log$=taxrep&RID=CPFJFVVV014) |
|  | TsDGAT2B | [PNH12928](https://www.ncbi.nlm.nih.gov/protein/PNH12928?report=genbank&log$=taxrep&RID=CPFF15Y7014) |
| *Xenopus tropicalis* | XtDGAT1 | [XP_031759943](https://www.ncbi.nlm.nih.gov/protein/XP_031759943?report=genbank&log$=taxrep&RID=CPE3WA6R014) |
| *Zea mays* | ZmWS1 | ONM18562.1 |
